# Bis{μ-2-[(pyrimidin-2-yl)amino­meth­yl]phenolato}-κ^2^ *N* ^1^:*O*;κ^2^ *O*:*N* ^1^-bis­({2-[(pyrimidin-2-yl-κ*N*)amino­meth­yl]phenol}silver(I)) dihydrate

**DOI:** 10.1107/S1600536812045783

**Published:** 2012-11-28

**Authors:** Shan Gao, Seik Weng Ng

**Affiliations:** a Key Laboratory of Functional Inorganic Material Chemistry, Ministry of Education, Heilongjiang University, Harbin 150080, People’s Republic of China; b Department of Chemistry, University of Malaya, 50603 Kuala Lumpur, Malaysia; c Chemistry Department, Faculty of Science, King Abdulaziz University, PO Box 80203 Jeddah, Saudi Arabia

## Abstract

The Ag^I^ atom in the title centrosymmetric dinuclear compound, [Ag_2_(C_11_H_10_N_3_O)_2_(C_11_H_11_N_3_O)_2_]·2H_2_O, shows a *T*-shaped coordination arising from bonding to the N atom of a neutral 2-[(pyrimidin-2-yl)amino­meth­yl]phenol ligand, the N atom of the 2-[(pyrimidin-2-yl)amino­meth­yl]phenolate anion [N—Ag—N = 171.8 (1)°] and the terminal O atom of the other anion [Ag—O = 2.606 (3) Å]. A pair of 2-[(pyrimidin-2-yl)amino­meth­yl]phenolate anions link the two Ag^I^ atoms to form the dinuclear compound. In the crystal, adjacent dinuclear mol­ecules are linked to the lattice water mol­ecules, generating an O—H⋯O- and N—H⋯O-connected three-dimensional network. In the crystal, the hy­droxy H atom is disordered over two positions in a 1:1 ratio; one half-occupancy H atom is connected to one hy­droxy group, whereas the other half-occupancy H atom is connected to another hy­droxy group.

## Related literature

For the structure of 2-{[(pyrimidin-2-yl)amino]­meth­yl}phenol, see: Xu *et al.* (2011 ▶). 
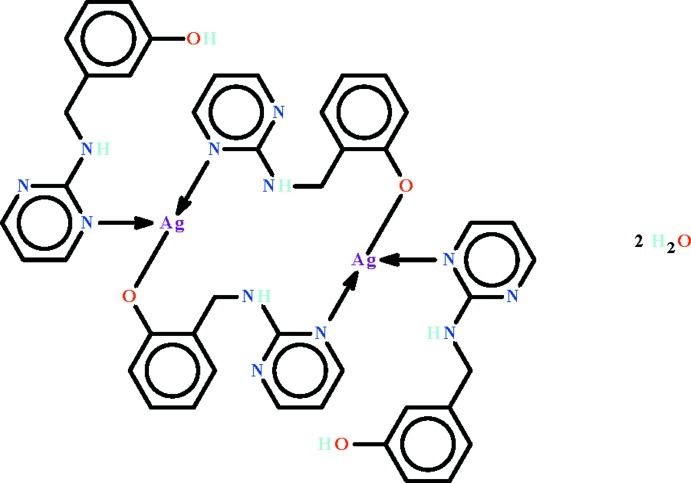


## Experimental

### 

#### Crystal data

[Ag_2_(C_11_H_10_N_3_O)_2_(C_11_H_11_N_3_O)_2_]·2H_2_O
*M* *_r_* = 1054.67
Monoclinic, 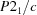

*a* = 9.2992 (4) Å
*b* = 24.808 (1) Å
*c* = 9.8158 (5) Å
β = 108.453 (1)°
*V* = 2148.02 (17) Å^3^
*Z* = 2
Mo *K*α radiation
μ = 0.98 mm^−1^
*T* = 293 K
0.23 × 0.20 × 0.17 mm

#### Data collection

Rigaku R-AXIS RAPID IP diffractometer
Absorption correction: multi-scan (*ABSCOR*; Higashi, 1995 ▶) *T*_min_ = 0.807, *T*_max_ = 0.852
20943 measured reflections
4910 independent reflections
3230 reflections with *I* > 2σ(*I*)
*R*_int_ = 0.063

#### Refinement

*R*[*F* ^2^ > 2σ(*F* ^2^)] = 0.044
*wR*(*F* ^2^) = 0.133
*S* = 1.11
4910 reflections
307 parameters
7 restraints
H atoms treated by a mixture of independent and constrained refinement
Δρ_max_ = 1.27 e Å^−3^
Δρ_min_ = −1.29 e Å^−3^

### 

Data collection: *RAPID-AUTO* (Rigaku, 1998 ▶); cell refinement: *RAPID-AUTO*; data reduction: *CrystalClear* (Rigaku/MSC, 2002 ▶); program(s) used to solve structure: *SHELXS97* (Sheldrick, 2008 ▶); program(s) used to refine structure: *SHELXL97* (Sheldrick, 2008 ▶); molecular graphics: *X-SEED* (Barbour, 2001 ▶); software used to prepare material for publication: *publCIF* (Westrip, 2010 ▶).

## Supplementary Material

Crystal structure: contains datablock(s) global, I. DOI: 10.1107/S1600536812045783/xu5644sup1.cif

Structure factors: contains datablock(s) I. DOI: 10.1107/S1600536812045783/xu5644Isup2.hkl

Additional supplementary materials:  crystallographic information; 3D view; checkCIF report

## Figures and Tables

**Table 1:** Hydrogen-bond geometry (Å, °)

*D*—H⋯*A*	*D*—H	H⋯*A*	*D*⋯*A*	*D*—H⋯*A*
O1—H1o⋯O2^i^	0.84 (1)	1.68 (3)	2.504 (4)	166 (12)
O2—H2o⋯O1^ii^	0.84 (1)	1.68 (3)	2.504 (4)	166 (13)
O1w—H11⋯O2^iii^	0.84 (1)	2.31 (6)	2.927 (5)	130 (6)
O1w—H12⋯N3^iv^	0.84 (1)	2.27 (2)	3.085 (5)	165 (6)
N1—H1⋯O1^v^	0.88 (1)	1.99 (1)	2.863 (5)	176 (4)
N6—H6⋯O1w	0.88 (1)	2.08 (2)	2.943 (5)	169 (5)

Symmetry codes: (i) 
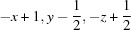
; (ii) 
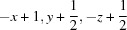
; (iii) 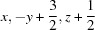; (iv) 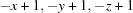; (v) 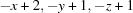.

**Fig. 1.**
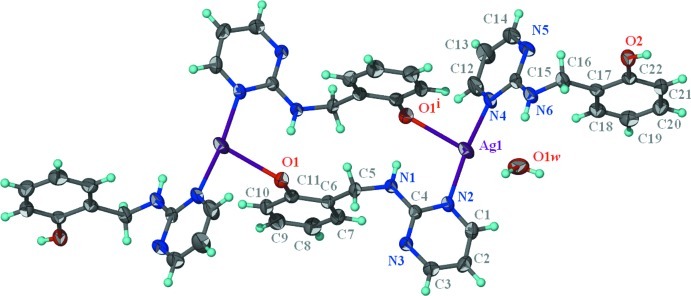
Thermal ellipsoid plot (Barbour, 2001) of [Ag2(C11H10N3O)2(C11H11N3O)]2.H2O at the 50% probability level; hydrogen atoms are drawn as spheres of arbitrary radius. The disorder in the phenolic –OH groups is not shown.

## supplementary crystallographic information

### Comment

A recent study reports 2-[(pyrimidin-2-yl)aminomethyl]phenol, a reduced Schiff-base that possesses an acidic phenolic group (Xu *et al.*, 2011). The reaction with silver nitrate yields dinuclear [Ag(C_11_H_10_N_3_O)(C_11_H_11_N_3_O)]_2_^.^2H_2_O (Scheme I, Fig. 1). The asymmetric unit has one Ag atom; however, the hydroxy H-atom is disordered over two positions in a 1:1 ratio. One half-occupancy H-atom is connected to O1 whereas the other half-occupancy atom is connected to O2. The Ag^I^ atom shows *T*-shaped coordination. Adjacent molecules are linked to the lattice water molecules to generate a O–H···O and N–H···O connected three-dimensional network (Table 1).

### Experimental

An acetonitrile solution (10 ml) of silver nitrate (1 mmol) was added to a methanol solution (5 ml) of 2-[(pyrimidin-2-yl)aminomethyl]phenol (1 mmol) and potassium hydroxide (0.5 mmol). The solution was filtered and then side aside, away from light, for the growth of crystals. Colorless crystals were obtained after several days.

### Refinement

Hydrogen atoms were placed in calculated positions (C–H 0.93–0.97 Å) and were included in the refinement in the riding model approximation, with *U*(H) set to 1.2*U*(C). The amino and hydroxy/water H atoms were located in a difference Fouier map, and were refined with distance restraints of N–H 0.88±0.01, O–H 0.84±0.01 and H···H 1.37±0.01 Å. Their temperature factors were refined.

The hydroxy H-atom is disordered over two positions in a 1:1 ratio; one half-occupancy H-atom is connected to O1 whereas the other half-occupancy atom is connected to O2.

The final difference Fouier map had a peak at 0.83 Å from Ag1 and a hole at 0.84 Å from this atom.

### Crystal data

**Table:**

[Ag_2_(C_11_H_10_N_3_O)_2_(C_11_H_11_N_3_O)_2_]·2H_2_O	*F*(000) = 1072
*M**_r_* = 1054.67	*D*_x_ = 1.631 Mg m^−^^3^
Monoclinic, *P*2_1_/*c*	Mo *K*α radiation, λ = 0.71073 Å
Hall symbol: -P 2ybc	Cell parameters from 13157 reflections
*a* = 9.2992 (4) Å	θ = 3.1–27.5°
*b* = 24.808 (1) Å	µ = 0.98 mm^−^^1^
*c* = 9.8158 (5) Å	*T* = 293 K
β = 108.453 (1)°	Prism, colorless
*V* = 2148.02 (17) Å^3^	0.23 × 0.20 × 0.17 mm
*Z* = 2	

### Data collection

**Table:**

Rigaku R-AXIS RAPID IP diffractometer	4910 independent reflections
Radiation source: fine-focus sealed tube	3230 reflections with *I* > 2σ(*I*)
Graphite monochromator	*R*_int_ = 0.063
ω scan	θ_max_ = 27.5°, θ_min_ = 3.1°
Absorption correction: multi-scan (*ABSCOR*; Higashi, 1995)	*h* = −12→11
*T*_min_ = 0.807, *T*_max_ = 0.852	*k* = −32→32
20943 measured reflections	*l* = −12→12

### Refinement

**Table:**

Refinement on *F*^2^	Primary atom site location: structure-invariant direct methods
Least-squares matrix: full	Secondary atom site location: difference Fourier map
*R*[*F*^2^ > 2σ(*F*^2^)] = 0.044	Hydrogen site location: inferred from neighbouring sites
*wR*(*F*^2^) = 0.133	H atoms treated by a mixture of independent and constrained refinement
*S* = 1.11	*w* = 1/[σ^2^(*F*_o_^2^) + (0.0382*P*)^2^ + 4.7243*P*] where *P* = (*F*_o_^2^ + 2*F*_c_^2^)/3
4910 reflections	(Δ/σ)_max_ = 0.001
307 parameters	Δρ_max_ = 1.27 e Å^−^^3^
7 restraints	Δρ_min_ = −1.29 e Å^−^^3^

### Fractional atomic coordinates and isotropic or equivalent isotropic displacement parameters (Å^2^)

**Table:**

	*x*	*y*	*z*	*U*_iso_*/*U*_eq_	Occ. (<1)
Ag1	0.70167 (5)	0.644035 (13)	0.56817 (5)	0.04905 (15)	
O1	1.0721 (3)	0.39077 (12)	0.5102 (3)	0.0383 (7)	
H1O	0.990 (7)	0.376 (4)	0.461 (10)	0.057*	0.50
O2	0.1768 (4)	0.84419 (14)	0.0994 (4)	0.0458 (8)	
H2O	0.095 (7)	0.858 (5)	0.050 (11)	0.069*	0.50
O1W	0.3708 (5)	0.64557 (15)	0.4170 (5)	0.0650 (11)	
H11	0.289 (4)	0.659 (2)	0.420 (8)	0.097*	
H12	0.353 (6)	0.6145 (12)	0.382 (7)	0.097*	
N1	0.8011 (4)	0.51659 (14)	0.5833 (4)	0.0371 (9)	
H1	0.838 (5)	0.5460 (11)	0.557 (5)	0.045*	
N2	0.6393 (4)	0.56969 (14)	0.6588 (4)	0.0362 (8)	
N3	0.6313 (4)	0.47353 (14)	0.6741 (4)	0.0404 (9)	
N4	0.7537 (4)	0.72440 (15)	0.5013 (5)	0.0441 (10)	
N5	0.7062 (5)	0.80270 (15)	0.3525 (5)	0.0482 (10)	
N6	0.5199 (4)	0.73932 (15)	0.3360 (5)	0.0441 (10)	
H6	0.487 (6)	0.7108 (13)	0.370 (5)	0.053*	
C1	0.5302 (5)	0.57248 (19)	0.7213 (6)	0.0451 (11)	
H1A	0.4953	0.6062	0.7375	0.054*	
C2	0.4682 (5)	0.5276 (2)	0.7621 (6)	0.0472 (12)	
H2A	0.3940	0.5301	0.8069	0.057*	
C3	0.5215 (6)	0.4788 (2)	0.7332 (6)	0.0482 (12)	
H3	0.4785	0.4477	0.7562	0.058*	
C4	0.6884 (5)	0.51959 (16)	0.6398 (5)	0.0328 (9)	
C5	0.8671 (5)	0.46637 (17)	0.5550 (5)	0.0350 (9)	
H5A	0.8979	0.4710	0.4701	0.042*	
H5B	0.7897	0.4386	0.5339	0.042*	
C6	1.0024 (5)	0.44711 (17)	0.6770 (5)	0.0350 (9)	
C7	1.0368 (6)	0.4667 (2)	0.8155 (5)	0.0443 (11)	
H7	0.9729	0.4919	0.8365	0.053*	
C8	1.1647 (6)	0.4494 (2)	0.9240 (6)	0.0533 (13)	
H8	1.1873	0.4635	1.0162	0.064*	
C9	1.2577 (6)	0.4113 (2)	0.8933 (6)	0.0499 (12)	
H9	1.3435	0.3995	0.9653	0.060*	
C10	1.2245 (5)	0.39041 (18)	0.7566 (5)	0.0404 (10)	
H10	1.2873	0.3641	0.7384	0.048*	
C11	1.0981 (5)	0.40809 (16)	0.6445 (5)	0.0347 (9)	
C12	0.8956 (6)	0.7416 (2)	0.5634 (7)	0.0660 (17)	
H12A	0.9608	0.7209	0.6360	0.079*	
C13	0.9484 (7)	0.7889 (2)	0.5233 (9)	0.078 (2)	
H13	1.0475	0.8005	0.5669	0.094*	
C14	0.8486 (6)	0.8178 (2)	0.4167 (7)	0.0580 (14)	
H14	0.8819	0.8498	0.3875	0.070*	
C15	0.6627 (5)	0.75617 (17)	0.3970 (5)	0.0392 (10)	
C16	0.4023 (6)	0.77407 (18)	0.2440 (5)	0.0446 (11)	
H16A	0.3197	0.7516	0.1869	0.054*	
H16B	0.4437	0.7930	0.1783	0.054*	
C17	0.3390 (5)	0.81517 (17)	0.3238 (5)	0.0355 (10)	
C18	0.3894 (6)	0.82048 (19)	0.4713 (5)	0.0447 (11)	
H18	0.4671	0.7983	0.5255	0.054*	
C19	0.3273 (6)	0.8580 (2)	0.5401 (6)	0.0502 (12)	
H19	0.3628	0.8609	0.6396	0.060*	
C20	0.2126 (6)	0.8911 (2)	0.4609 (6)	0.0503 (12)	
H20	0.1701	0.9163	0.5069	0.060*	
C21	0.1606 (6)	0.88692 (19)	0.3134 (6)	0.0462 (12)	
H21	0.0834	0.9095	0.2604	0.055*	
C22	0.2225 (5)	0.84915 (17)	0.2430 (5)	0.0361 (10)	

### Atomic displacement parameters (Å^2^)

**Table:**

	*U*^11^	*U*^22^	*U*^33^	*U*^12^	*U*^13^	*U*^23^
Ag1	0.0552 (3)	0.02896 (19)	0.0589 (3)	0.00169 (16)	0.01237 (19)	0.00829 (16)
O1	0.0365 (17)	0.0369 (16)	0.0407 (19)	−0.0016 (14)	0.0111 (15)	−0.0062 (13)
O2	0.047 (2)	0.0487 (19)	0.0391 (19)	0.0124 (16)	0.0096 (16)	0.0051 (15)
O1W	0.076 (3)	0.048 (2)	0.081 (3)	−0.020 (2)	0.040 (3)	−0.005 (2)
N1	0.036 (2)	0.0258 (17)	0.054 (2)	0.0002 (15)	0.0206 (19)	0.0032 (16)
N2	0.0352 (19)	0.0296 (17)	0.042 (2)	0.0038 (16)	0.0092 (17)	−0.0002 (15)
N3	0.040 (2)	0.0309 (18)	0.055 (3)	0.0011 (16)	0.0223 (19)	0.0027 (17)
N4	0.036 (2)	0.0319 (19)	0.061 (3)	0.0074 (17)	0.011 (2)	0.0106 (18)
N5	0.054 (3)	0.036 (2)	0.055 (3)	0.0006 (19)	0.018 (2)	0.0101 (18)
N6	0.042 (2)	0.0288 (19)	0.058 (3)	0.0006 (17)	0.010 (2)	0.0057 (18)
C1	0.043 (3)	0.040 (2)	0.053 (3)	0.009 (2)	0.018 (2)	−0.003 (2)
C2	0.035 (2)	0.060 (3)	0.050 (3)	0.006 (2)	0.019 (2)	0.000 (2)
C3	0.044 (3)	0.043 (3)	0.062 (3)	−0.002 (2)	0.023 (3)	0.007 (2)
C4	0.029 (2)	0.028 (2)	0.039 (2)	−0.0002 (17)	0.0069 (19)	0.0012 (17)
C5	0.030 (2)	0.031 (2)	0.046 (3)	0.0026 (18)	0.014 (2)	−0.0020 (18)
C6	0.035 (2)	0.032 (2)	0.040 (2)	−0.0017 (18)	0.016 (2)	0.0007 (18)
C7	0.047 (3)	0.045 (3)	0.044 (3)	0.007 (2)	0.020 (2)	0.000 (2)
C8	0.056 (3)	0.062 (3)	0.040 (3)	0.004 (3)	0.014 (3)	−0.001 (2)
C9	0.044 (3)	0.059 (3)	0.045 (3)	0.005 (2)	0.011 (2)	0.009 (2)
C10	0.035 (2)	0.038 (2)	0.048 (3)	0.007 (2)	0.012 (2)	0.003 (2)
C11	0.036 (2)	0.030 (2)	0.041 (3)	−0.0045 (18)	0.015 (2)	−0.0016 (18)
C12	0.042 (3)	0.052 (3)	0.095 (5)	0.010 (3)	0.008 (3)	0.022 (3)
C13	0.040 (3)	0.058 (4)	0.131 (6)	−0.005 (3)	0.017 (4)	0.022 (4)
C14	0.053 (3)	0.044 (3)	0.079 (4)	−0.004 (3)	0.024 (3)	0.010 (3)
C15	0.042 (3)	0.028 (2)	0.050 (3)	0.0072 (19)	0.019 (2)	0.0009 (19)
C16	0.044 (3)	0.033 (2)	0.053 (3)	0.005 (2)	0.010 (2)	0.002 (2)
C17	0.036 (2)	0.030 (2)	0.039 (2)	−0.0042 (18)	0.009 (2)	0.0043 (18)
C18	0.045 (3)	0.041 (2)	0.047 (3)	−0.004 (2)	0.013 (2)	0.004 (2)
C19	0.060 (3)	0.054 (3)	0.036 (3)	−0.007 (3)	0.013 (2)	−0.003 (2)
C20	0.055 (3)	0.051 (3)	0.051 (3)	0.002 (2)	0.025 (3)	−0.007 (2)
C21	0.045 (3)	0.040 (3)	0.057 (3)	0.007 (2)	0.021 (3)	0.004 (2)
C22	0.033 (2)	0.035 (2)	0.041 (2)	−0.0045 (18)	0.013 (2)	0.0049 (18)

### Geometric parameters (Å, º)

**Table:**

Ag1—N4	2.200 (4)	C5—H5A	0.9700
Ag1—N2	2.204 (4)	C5—H5B	0.9700
Ag1—O1^i^	2.606 (3)	C6—C7	1.382 (6)
Ag1—O1W	2.963 (5)	C6—C11	1.419 (6)
O1—C11	1.333 (5)	C7—C8	1.389 (7)
O1—H1O	0.840 (10)	C7—H7	0.9300
O2—C22	1.343 (5)	C8—C9	1.379 (7)
O2—H2O	0.839 (10)	C8—H8	0.9300
O1W—H11	0.842 (10)	C9—C10	1.379 (7)
O1W—H12	0.840 (10)	C9—H9	0.9300
N1—C4	1.334 (5)	C10—C11	1.402 (6)
N1—C5	1.454 (5)	C10—H10	0.9300
N1—H1	0.877 (10)	C12—C13	1.376 (8)
N2—C1	1.343 (6)	C12—H12A	0.9300
N2—C4	1.357 (5)	C13—C14	1.363 (8)
N3—C3	1.331 (6)	C13—H13	0.9300
N3—C4	1.347 (5)	C14—H14	0.9300
N4—C12	1.336 (7)	C16—C17	1.514 (6)
N4—C15	1.356 (6)	C16—H16A	0.9700
N5—C14	1.328 (7)	C16—H16B	0.9700
N5—C15	1.341 (6)	C17—C18	1.379 (6)
N6—C15	1.340 (6)	C17—C22	1.403 (6)
N6—C16	1.459 (6)	C18—C19	1.379 (7)
N6—H6	0.877 (10)	C18—H18	0.9300
C1—C2	1.371 (7)	C19—C20	1.375 (7)
C1—H1A	0.9300	C19—H19	0.9300
C2—C3	1.371 (7)	C20—C21	1.377 (7)
C2—H2A	0.9300	C20—H20	0.9300
C3—H3	0.9300	C21—C22	1.393 (6)
C5—C6	1.514 (6)	C21—H21	0.9300
			
N4—Ag1—N2	171.75 (14)	C9—C8—C7	119.2 (5)
N4—Ag1—O1^i^	86.43 (12)	C9—C8—H8	120.4
N2—Ag1—O1^i^	100.50 (11)	C7—C8—H8	120.4
N4—Ag1—O1W	97.51 (13)	C10—C9—C8	120.5 (5)
N2—Ag1—O1W	81.30 (12)	C10—C9—H9	119.7
O1^i^—Ag1—O1W	131.60 (11)	C8—C9—H9	119.7
C11—O1—H1O	124 (8)	C9—C10—C11	121.4 (4)
C22—O2—H2O	120 (9)	C9—C10—H10	119.3
Ag1—O1W—H11	142 (4)	C11—C10—H10	119.3
Ag1—O1W—H12	103 (4)	O1—C11—C10	121.3 (4)
H11—O1W—H12	109 (2)	O1—C11—C6	120.8 (4)
C4—N1—C5	124.2 (3)	C10—C11—C6	117.8 (4)
C4—N1—H1	120 (3)	N4—C12—C13	122.1 (5)
C5—N1—H1	115 (3)	N4—C12—H12A	118.9
C1—N2—C4	116.5 (4)	C13—C12—H12A	118.9
C1—N2—Ag1	118.3 (3)	C14—C13—C12	116.8 (5)
C4—N2—Ag1	124.6 (3)	C14—C13—H13	121.6
C3—N3—C4	116.3 (4)	C12—C13—H13	121.6
C12—N4—C15	116.7 (4)	N5—C14—C13	123.3 (5)
C12—N4—Ag1	115.6 (3)	N5—C14—H14	118.3
C15—N4—Ag1	127.5 (3)	C13—C14—H14	118.3
C14—N5—C15	116.5 (4)	N6—C15—N5	118.8 (4)
C15—N6—C16	122.4 (4)	N6—C15—N4	116.8 (4)
C15—N6—H6	120 (4)	N5—C15—N4	124.5 (4)
C16—N6—H6	115 (4)	N6—C16—C17	114.6 (4)
N2—C1—C2	122.7 (4)	N6—C16—H16A	108.6
N2—C1—H1A	118.7	C17—C16—H16A	108.6
C2—C1—H1A	118.7	N6—C16—H16B	108.6
C3—C2—C1	116.3 (4)	C17—C16—H16B	108.6
C3—C2—H2A	121.8	H16A—C16—H16B	107.6
C1—C2—H2A	121.8	C18—C17—C22	118.8 (4)
N3—C3—C2	123.7 (4)	C18—C17—C16	123.3 (4)
N3—C3—H3	118.2	C22—C17—C16	118.0 (4)
C2—C3—H3	118.2	C19—C18—C17	121.5 (5)
N1—C4—N3	118.7 (4)	C19—C18—H18	119.3
N1—C4—N2	116.8 (4)	C17—C18—H18	119.3
N3—C4—N2	124.4 (4)	C20—C19—C18	119.7 (5)
N1—C5—C6	114.5 (4)	C20—C19—H19	120.1
N1—C5—H5A	108.6	C18—C19—H19	120.1
C6—C5—H5A	108.6	C19—C20—C21	120.1 (5)
N1—C5—H5B	108.6	C19—C20—H20	120.0
C6—C5—H5B	108.6	C21—C20—H20	120.0
H5A—C5—H5B	107.6	C20—C21—C22	120.6 (5)
C7—C6—C11	119.6 (4)	C20—C21—H21	119.7
C7—C6—C5	122.9 (4)	C22—C21—H21	119.7
C11—C6—C5	117.4 (4)	O2—C22—C21	122.6 (4)
C6—C7—C8	121.5 (4)	O2—C22—C17	118.0 (4)
C6—C7—H7	119.3	C21—C22—C17	119.3 (4)
C8—C7—H7	119.3		
			
O1^i^—Ag1—N2—C1	175.4 (3)	C7—C6—C11—O1	−176.3 (4)
O1W—Ag1—N2—C1	−53.7 (3)	C5—C6—C11—O1	2.4 (6)
O1^i^—Ag1—N2—C4	−13.9 (4)	C7—C6—C11—C10	0.7 (6)
O1W—Ag1—N2—C4	117.0 (4)	C5—C6—C11—C10	179.4 (4)
O1^i^—Ag1—N4—C12	−55.8 (4)	C15—N4—C12—C13	−0.6 (9)
O1W—Ag1—N4—C12	172.7 (4)	Ag1—N4—C12—C13	174.3 (5)
O1^i^—Ag1—N4—C15	118.4 (4)	N4—C12—C13—C14	0.2 (11)
O1W—Ag1—N4—C15	−13.1 (4)	C15—N5—C14—C13	−0.4 (9)
C4—N2—C1—C2	−1.1 (7)	C12—C13—C14—N5	0.4 (10)
Ag1—N2—C1—C2	170.3 (4)	C16—N6—C15—N5	−12.2 (7)
N2—C1—C2—C3	−1.2 (8)	C16—N6—C15—N4	167.5 (4)
C4—N3—C3—C2	−1.3 (8)	C14—N5—C15—N6	179.7 (5)
C1—C2—C3—N3	2.6 (8)	C14—N5—C15—N4	−0.1 (7)
C5—N1—C4—N3	0.4 (7)	C12—N4—C15—N6	−179.2 (5)
C5—N1—C4—N2	−179.7 (4)	Ag1—N4—C15—N6	6.6 (6)
C3—N3—C4—N1	178.5 (4)	C12—N4—C15—N5	0.6 (7)
C3—N3—C4—N2	−1.4 (7)	Ag1—N4—C15—N5	−173.6 (3)
C1—N2—C4—N1	−177.3 (4)	C15—N6—C16—C17	−77.1 (6)
Ag1—N2—C4—N1	11.8 (6)	N6—C16—C17—C18	1.2 (6)
C1—N2—C4—N3	2.6 (7)	N6—C16—C17—C22	−178.3 (4)
Ag1—N2—C4—N3	−168.3 (3)	C22—C17—C18—C19	0.5 (7)
C4—N1—C5—C6	−91.6 (5)	C16—C17—C18—C19	−179.1 (4)
N1—C5—C6—C7	16.3 (6)	C17—C18—C19—C20	−0.1 (7)
N1—C5—C6—C11	−162.4 (4)	C18—C19—C20—C21	−0.3 (8)
C11—C6—C7—C8	0.8 (7)	C19—C20—C21—C22	0.4 (7)
C5—C6—C7—C8	−177.8 (4)	C20—C21—C22—O2	−179.1 (4)
C6—C7—C8—C9	−1.2 (8)	C20—C21—C22—C17	0.0 (7)
C7—C8—C9—C10	0.1 (8)	C18—C17—C22—O2	178.7 (4)
C8—C9—C10—C11	1.5 (7)	C16—C17—C22—O2	−1.7 (6)
C9—C10—C11—O1	175.2 (4)	C18—C17—C22—C21	−0.4 (6)
C9—C10—C11—C6	−1.9 (6)	C16—C17—C22—C21	179.2 (4)

Symmetry code: (i) −*x*+2, −*y*+1, −*z*+1.

### Hydrogen-bond geometry (Å, º)

**Table:**

*D*—H···*A*	*D*—H	H···*A*	*D*···*A*	*D*—H···*A*
O1—H1o···O2^ii^	0.84 (1)	1.68 (3)	2.504 (4)	166 (12)
O2—H2o···O1^iii^	0.84 (1)	1.68 (3)	2.504 (4)	166 (13)
O1w—H11···O2^iv^	0.84 (1)	2.31 (6)	2.927 (5)	130 (6)
O1w—H12···N3^v^	0.84 (1)	2.27 (2)	3.085 (5)	165 (6)
N1—H1···O1^i^	0.88 (1)	1.99 (1)	2.863 (5)	176 (4)
N6—H6···O1w	0.88 (1)	2.08 (2)	2.943 (5)	169 (5)

Symmetry codes: (i) −*x*+2, −*y*+1, −*z*+1; (ii) −*x*+1, *y*−1/2, −*z*+1/2; (iii) −*x*+1, *y*+1/2, −*z*+1/2; (iv) *x*, −*y*+3/2, *z*+1/2; (v) −*x*+1, −*y*+1, −*z*+1.
